# A Reversal in Hair Cell Orientation Organizes Both the Auditory and Vestibular Organs

**DOI:** 10.3389/fnins.2021.695914

**Published:** 2021-09-27

**Authors:** Basile Tarchini

**Affiliations:** ^1^The Jackson Laboratory, Bar Harbor, ME, United States; ^2^Department of Medicine, Tufts University, Boston, MA, United States; ^3^Graduate School of Biomedical Science and Engineering (GSBSE), University of Maine, Orono, ME, United States

**Keywords:** cell polarity, hair cell, otolith organ, cochlea, neuromast, stereocilia bundle, hearing, balance

## Abstract

Sensory hair cells detect mechanical stimuli with their hair bundle, an asymmetrical brush of actin-based membrane protrusions, or stereocilia. At the single cell level, stereocilia are organized in rows of graded heights that confer the hair bundle with intrinsic directional sensitivity. At the organ level, each hair cell is precisely oriented so that its intrinsic directional sensitivity matches the direction of mechanical stimuli reaching the sensory epithelium. Coordinated orientation among neighboring hair cells usually ensures the delivery of a coherent local group response. Accordingly, hair cell orientation is locally uniform in the auditory and vestibular cristae epithelia in birds and mammals. However, an exception to this rule is found in the vestibular macular organs, and in fish lateral line neuromasts, where two hair cell populations show opposing orientations. This mirror-image hair cell organization confers bidirectional sensitivity at the organ level. Here I review our current understanding of the molecular machinery that produces mirror-image organization through a regional reversal of hair cell orientation. Interestingly, recent evidence suggests that auditory hair cells adopt their normal uniform orientation through a global reversal mechanism similar to the one at work regionally in macular and neuromast organs. Macular and auditory organs thus appear to be patterned more similarly than previously appreciated during inner ear development.

## Introduction

**FIGURE 1:**
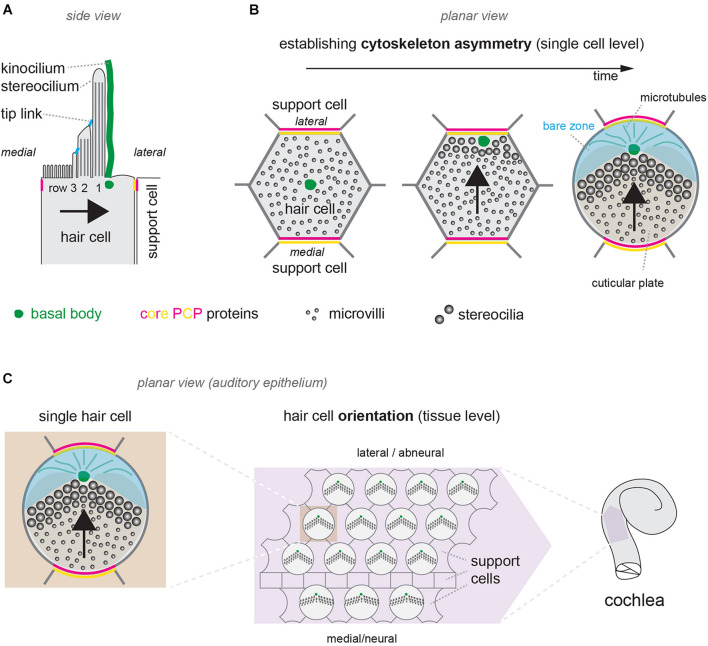
Cytoskeleton asymmetry in single hair cells and hair cell orientation at the tissue level. **(A)** Diagram of the apical surface of a single mouse neonate auditory HC (IHC). Cytoskeleton asymmetry in this side view includes stereocilia with graded heights by row, with the tallest row 1 on the side of the off-center basal body that nucleates the kinocilium (green). **(B)** Planar (en-face) view illustrating symmetry breaking and cytoskeleton asymmetry in a single developing auditory HC during late embryogenesis. The basal body (green) shifts off-center toward the lateral HC junction. Microvilli on the side of the off-center basal body grow in diameter and height to become stereocilia and form the hair bundle. With time, a bare zone deprived of microvilli (blue) emerges between the basal body and the lateral HC junction. In panels **(A,B)** segregation of the two distinct core PCP complexes in single HCs and their juxtaposition at the apical HC-support cell junctions are represented in magenta and yellow (see main text). **(C)** Diagram showing the orientation of auditory HCs in the sensory epithelium around birth. Each HC orients its planar-asymmetric apical cytoskeleton so that the off-center basal body (green) and V-shaped hair bundle point toward the lateral (abneural) side of the auditory epithelium. In all panels, arrows indicate HC orientation based on the position of the basal body/kinocilium, the shape of the hair bundle and other planar-asymmetric cytoskeletal elements.

The reception and transmission of mechanical stimuli by sensory hair cells (HCs) underlies the ability to hear and to perceive self and environmental motions. Mechanical stimuli range from sound waves in the auditory organ [the cochlea (Schwander et al., 2010)], to head movements and gravity in the balance organs [the vestibular system (Eatock and Songer, 2011)], to water movements in the lateral line system of fish and amphibians (Chitnis et al., 2012). The transduction of physical movements into biological signals occurs in the hair bundle, a critical apical compartment common to all HC types (McGrath et al., 2017; Velez-Ortega and Frolenkov, 2019). Depending on organ type and HC location within the organ, hair bundles differ in the number, dimensions and organization of their individual membrane protrusions, or stereocilia (Barr-Gillespie, 2015). However, hair bundles conform to some fundamental shared principles. Their stereocilia are supported by an F-actin paracrystal core and are always aligned in multiple rows of graded heights. This slanted, asymmetrical architecture is integral to the directional response of the hair bundle: only deflections of the hair bundle toward the tallest row produce optimal tension on tip links connecting rows of different heights (Figure 1A). In turn, this tension favorably influences the opening probability of ion channels located at the lower end of each tip link (Qiu and Muller, 2018; Zheng and Holt, 2021). The influx of ions in stereocilia generates a receptor potential which leads to the depolarization of the HC.

## Establishing and Orienting an Asymmetrical Cytoskeleton: Two Distinct Polarity Features Shared by All Hair Cells

At the single cell level, polarization starts as a break of cytoskeleton symmetry in young post-mitotic HCs. First, the roughly central basal body and the primary cilium it nucleates, termed the kinocilium, shift off-center (Figure 1B) (reviewed previously, see for example Deans, 2013; Tarchini and Lu, 2019; Montcouquiol and Kelley, 2020). Of note, the kinocilium is the only true, microtubule-based cilium in HCs. Via still unknown mechanisms, microvilli in the vicinity of the off-center basal body grow in diameter and height to become stable stereocilia. In mouse auditory HCs, stereocilia are corralled by the emergence and expansion of a smooth region of apical membrane between the shifted basal body and the lateral/abneural HC junction, the “bare zone” (Figure 1B). The bare zone and molecular links connecting central stereocilia to the kinocilium impart an asymmetrical V-shaped or semicircular edge to the forming hair bundle (Figure 1B). Stereocilia become precisely aligned, and the most lateral stereocilia abutting the bare zone grow into the tallest row. Under the apical surface, the hair bundle becomes supported by a pedestal of dense actin meshwork called the cuticular plate (Figure 1B). The cuticular plate is itself asymmetrical, as it accommodates the basal body on the bare zone side. Apical microtubules are asymmetrically distributed, because they are excluded and constrained to the basal body side by the cuticular plate. In summary, the hair bundle and external structures like the basal body, cuticular plate and microtubules are intimately interconnected, and become globally planar-polarized. This polarization process occurs at the single cell level, and will confer the hair bundle with an intrinsic directional sensitivity.

The hair bundle can be compared to an antenna. Proper signal detection requires an antenna to be intrinsically sensitive to signal direction, but also to be oriented correctly relative to the source of the signal. A complicated aspect of early HC polarization is that the generation of an asymmetrical cytoskeleton, summarized above, implicitly defines an orientation for each HC at the organ level. Symmetry breaking occurs in a single cell as the kinocilium moves off-center, but the direction of this move at the organ level (i.e., toward the lateral edge of the auditory epithelium in cochlear HCs; Figure 1C) also provides the HC with its initial orientation. Establishing cytoskeleton asymmetry in single HCs and orienting the resulting structures at the tissue level may thus appear to be the same polarization process. However, several lines of evidence show that these are, in fact, distinct processes.

The first line of evidence is that HCs can be misoriented from their earliest stage of differentiation and still develop a normal hair bundle and apical cytoskeleton. This was documented in core planar cell polarity (PCP) mutants such as *Vangl2* and double *Fzd3,6* mutants (Montcouquiol et al., 2003; Wang et al., 2006; Song et al., 2010). Core PCP proteins form conserved apical junction complexes that are required for intercellular communication and uniform local cell orientation (Goodrich and Strutt, 2011; Singh and Mlodzik, 2012; Butler and Wallingford, 2017). The VANGL2 and FZD3,6 complexes antagonize and exclude each other inside a single HC or support cell, but they have high affinity for each other in the extracellular space, relaying polarity information across cell neighbors to coordinate their orientation. For example, FZD3,6 located medially in a HC interacts with VANGL2 located laterally in the adjacent support cell (Figures 1A,B). Core PCP and its role in patterning the inner ear has been reviewed extensively (see for example Deans, 2013; Tarchini and Lu, 2019; Montcouquiol and Kelley, 2020), and a companion article by Deans and colleagues in this issue provides a useful update. Two results in the core PCP field are particularly worth a mention here. First, core PCP information is already propagated across precursors of HC and support cells (Wang et al., 2005; Montcouquiol et al., 2006), and asymmetrical PCP complexes are observed prior to symmetry breaking in HCs (Figure 1B; Deans et al., 2007; Jones et al., 2008). Second, the aberrant position of the basal body following its early off-center shift in the *Vangl2* mutant HCs foretells the pattern of HC misorientation observed at later stages when the hair bundle is differentiated (Montcouquiol et al., 2003). The evidence thus suggests that core PCP proteins provide an early junctional framework throughout the sensory epithelium. This framework instructs the orientation of the early basal body shift in HCs, and by extension, the orientation of the whole HC apical cytoskeleton (Figure 1B). Of note, however, core PCP proteins are not required for the shift itself, only for defining its orientation.

A second line of evidence for distinct polarization processes establishing and orienting the asymmetrical cytoskeleton is that HCs with a severely dysmorphic apical cytoskeleton can adopt a largely normal orientation (as judged by the position of the basal body/kinocilium, for example). USHER1 proteins form transient fibrous links interconnecting emerging stereocilia, and the resulting loss of stereocilia cohesion in *Usher1* mutants can give rise to dramatically misshapen hair bundles that lack a distinct V-shape or graded stereocilia heights (Lefevre et al., 2008; Webb et al., 2011). Nevertheless, auditory HCs in *Usher1* mutants are overall oriented laterally based on the position of other apical cytoskeletal elements. It is important to note, however, that misplaced stereocilia will disrupt the precise positioning of physically linked cytoskeletal elements, for example the kinocilium and its associated basal body (Webb et al., 2011). Although the resulting apical HC defects have frequently been described as HC misorientation (PCP phenotype), the low magnitude of the purported misorientation in comparison to core PCP phenotypes suggests otherwise. In other words, defective morphogenesis in single HCs may be sufficient to account for their mild apparent “misorientation.” This conclusion is supported by multiple observations of mildly mispositioned stereocilia or kinocilium when cell adhesion (for example Fukuda et al., 2014) or cell fate determination (for example Kiernan et al., 2005; Zhang et al., 2017) is altered. In these cases, disruptions in the orderly mosaic between HCs and support cells alter junctional tension and might indirectly cause stereocilia and kinocilium mispositioning in HCs.

Another example is provided by a protein complex that forms the bare zone: the inhibitory G proteins (Gαi1-3), the scaffolding protein GPSM2 and the adaptor INSC. Unlike USHER1 proteins, Gαi-GPSM2-INSC is planar-polarized, and occupies the HC apical membrane but not the apical HC junction with neighboring support cells, where core PCP proteins reside (Figure 1B). Loss of Gαi3, GPSM2 or INSC variably disrupts stereocilia positioning while also upsetting the position of the basal body/kinocilium, as in *Usher1* mutants. However, auditory HCs also maintain a generally lateral orientation in *Gαi3*, *Gpsm2* or *Insc* mutants (Ezan et al., 2013; Tarchini et al., 2013; Bhonker et al., 2016).

Finally, evidence that distinct polarization processes establish and orient the asymmetrical cytoskeleton was also obtained at later stages of HC differentiation. While the apical cytoskeleton maintains its asymmetry in maturing HCs, HC orientation is not strictly fixed in time. First, following the early off-center shift of the basal body, HC orientation is refined and becomes more precisely lateral in the cochlea with time (Dabdoub et al., 2003). Second, severe auditory HC misorientation in *Vangl2* and *Fzd3,6* mutants is corrected to a large extent after birth by a still unknown mechanism independent from core PCP (Copley et al., 2013; Nemelka et al., 2020, PS#897). Just as the orientation of an antenna can be adjusted to better capture external signals, the entire HC apical cytoskeleton is rotated during orientation correction, including the hair bundle and external structures. It remains unclear whether the whole HC (including its baso-lateral plasma membrane) is rotated, or only surface structures past the apical junctions.

## A Regional Reversal in Hair Cell Orientation Produces Mirror-Image Anatomy in Macular and Neuromast Organs

**FIGURE 2:**
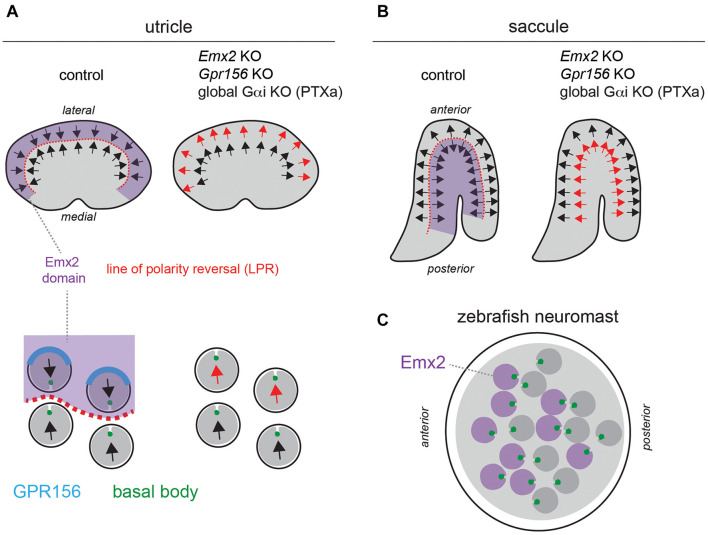
A reversal in hair cell orientation occurs in the macular organs of the vestibular system and in fish neuromasts. **(A,B)** Diagram of HC orientation (arrows) and *Emx2* regional expression (purple) in the mouse utricular **(A)** and saccular **(B)** maculae. A regional reversal in HC orientation in the *Emx2*-positive lateral utricle **(A)** and posterior saccule **(B)** creates a mirror-image organization at the organ level. This organization is lost in *Emx2* and *Gpr156* mutants, as well as upon Pertussis toxin (PTXa) expression. The bottom diagrams in panel **(A)** (utricle) show polarized enrichment of GPR156 (blue) at the lateral HC junction in *Emx2*-positive HCs above the line of polarity reversal (LPR, red), but not in *Emx2*-negative HCs below the LPR. **(C)** Diagram of an anterior-posterior neuromast in the larval zebrafish posterior lateral line. HCs expressing *emx2* are indicated in purple. In all panels, arrows indicate HC orientation based on the position of the basal body/kinocilium (green), the shape of the hair bundle and other planar-asymmetric cytoskeletal elements.

Establishing an asymmetrical cytoskeleton and defining its orientation across neighboring HCs describes the polarization process in avian and mammalian auditory epithelia and in semicircular canal cristae, where HCs detect angular acceleration. However, these mechanisms do not account for an intriguing conserved feature in macular (otolith) organs detecting linear acceleration and gravity, and in fish neuromasts sensing water movements. In maculae and neuromasts, HCs are sorted into two populations with opposing orientations (Flock and Wersall, 1962; Flock, 1964; Lindeman, 1969). HC orientation in the maculae varies across the organ in order to capture head movements in a range of orientations within an approximately horizontal (utricle) or vertical (saccule) plane (Figures 2A,B). However, this gradual change is distinct from an abrupt change in HC orientation that is observed across a virtual line of polarity reversal (LPR; Figures 2A,B; Li et al., 2008). In fish neuromasts, progenitors undergo a final division and sibling HCs acquire opposing orientations (Lopez-Schier et al., 2004; Chitnis et al., 2012). As a result, neuromasts have two equal populations of HCs with mirror-image orientations, but there is no continuous LPR (Figure 2C).

Mirror-image organization implies that opposing HCs in the same organ produce opposite responses to the same stimulus: HC depolarization and increased afferent spike rate on one side, and HC hyperpolarization and decreased spike rate on the other side (Harada et al., 1984; Lu et al., 1998; Lu and Popper, 2001). Mirror-image HC organization endows each neuromast with bidirectional sensitivity to detect predators and prey, for rheotaxis and for schooling behavior (Lopez-Schier et al., 2004; Ghysen and Dambly-Chaudiere, 2007; Chitnis et al., 2012). In contrast, the role of mirror-image HC organization in macular organs remains uncertain. It may complement the incomplete range of HC orientation on either side of the LPR to achieve 360° sensitivity, and/or be a strategy to enhance evoked activity in downstream neurons by combining positive and negative signals.

A critical observation is that the pattern of core PCP protein enrichment is unchanged across the LPR in the macular organs, and thus cannot by itself instruct a reversal in HC orientation in one half of the organ (Deans et al., 2007; Jones et al., 2014). Zebrafish neuromasts similarly show uniform enrichment of core PCP proteins regardless of HC orientation (Mirkovic et al., 2012). This led to the conclusion that asymmetric PCP cues at apical junctions likely act as a framework that can be independently interpreted by other factors in HCs (Deans et al., 2007). Such factors would consequently differ across the LPR, or be limited to one side.

One obvious candidate emerged from studies investigating the gene mutated in the deaf mouse mutant *Pardon*, the homeobox transcription factor *Emx2* (Rhodes et al., 2003). Interestingly, *Emx2* loss-of-function abolishes mirror-image organization and the LPR in macular organs without affecting locally coordinated HC orientation, or the gradual change in HC orientation along the organ (Figures 2A,B; Holley et al., 2010). Relatedly, inactivating *emx2* in zebrafish produces neuromasts where a normal number of HCs all adopt the same orientation, leading to unidirectional sensitivity (Jiang et al., 2017).

Regional *Emx2* transcription can fully explain the loss of mirror-image HC organization in the vestibular system and in neuromasts in *Emx2* mutants. In the mouse maculae, *Emx2* transcripts are limited to the lateral utricle and posterior saccule, the specific regions where HCs are flipped by 180° in the mutants (Figures 2A,B). Therefore, *Emx2* normally functions to reverse HC orientation there compared to the rest of the macula, creating the LPR (Jiang et al., 2017). *Emx2* regional specificity to one side of the LPR is conserved in the macular organs of the chicken (Jiang et al., 2017). In neuromasts, *emx2* expression is similarly regional and limited to HCs that are flipped in the mutant: HCs that detect anterior (A) > posterior (P) flow in A-P neuromasts, and HCs that detect dorsal (D) > ventral (V) flow in D-V neuromasts (Figure 2C; Jiang et al., 2017). Remarkably, forcing *Emx2* expression in all macular or neuromast HCs prompts HCs that normally do not express *Emx2* to reverse their orientation (Jiang et al., 2017). This gain-of-function produces an organ where all HCs adopt a uniform orientation that is opposite from what is observed upon *Emx2* loss-of-function. In summary, *Emx2* is necessary and sufficient to reverse a ground state of HC orientation by 180°.

Logically, *Emx2* expression is absent in the cristae of the semicircular canals that do not harbor mirror-image HC organization (Holley et al., 2010; Jiang et al., 2017). Accordingly, HC orientation is unaltered in *Emx2* mutant cristae (Holley et al., 2010; Jiang et al., 2017).

Together, these regional expression patterns and phenotypes suggest that EMX2 effectors in HCs, perhaps as direct transcriptional target(s), reverse the interpretation of invariant core PCP cues at cell-cell junctions. At early stages, this could reverse the orientation of the basal body shift, which in turn would reverse the orientation of the whole asymmetrical HC cytoskeleton. Support for a reversed shift of the basal body across the LPR was indeed obtained using live-imaging of explanted utricles (Tona and Wu, 2020). Of note, these studies collectively demonstrate that HC orientation is not only regulated by intercellular mechanisms acting at the tissue level (core PCP), but also by a mechanism acting at the single cell level (Emx2-triggered reversal). Distinct HC-instrinsic mechanisms thus influence symmetry breaking (establishing cytoskeleton asymmetry) as well as HC orientation.

In zebrafish neuromasts, uniformly blocking or over-activating Notch signaling also biases HCs toward one orientation (Mirkovic et al., 2012; Dow et al., 2018), in addition to disturbing the production of new HCs from support cells (Haddon et al., 1998; Wibowo et al., 2011). The most recent evidence suggests that Notch-mediated lateral inhibition influences mirror-image HC organization in part by influencing *emx2* expression (Jacobo et al., 2019; Kozak et al., 2020).

Interestingly, *Emx2* is transcribed throughout the auditory epithelium in mouse (Holley et al., 2010; Jiang et al., 2017). A specific role for *Emx2* in auditory HC orientation is difficult to evaluate, however, because constitutive *Emx2* mutants lack outer HCs (OHCs). Inner HC (IHCs) are severely misaligned (Holley et al., 2010), and improper contacts with support cell neighbors may explain their imprecise orientation in *Emx2* mutants (Holley et al., 2010).

## A Global Reversal in Hair Cell Orientation Unexpectedly Shapes the Auditory Organ

**FIGURE 3:**
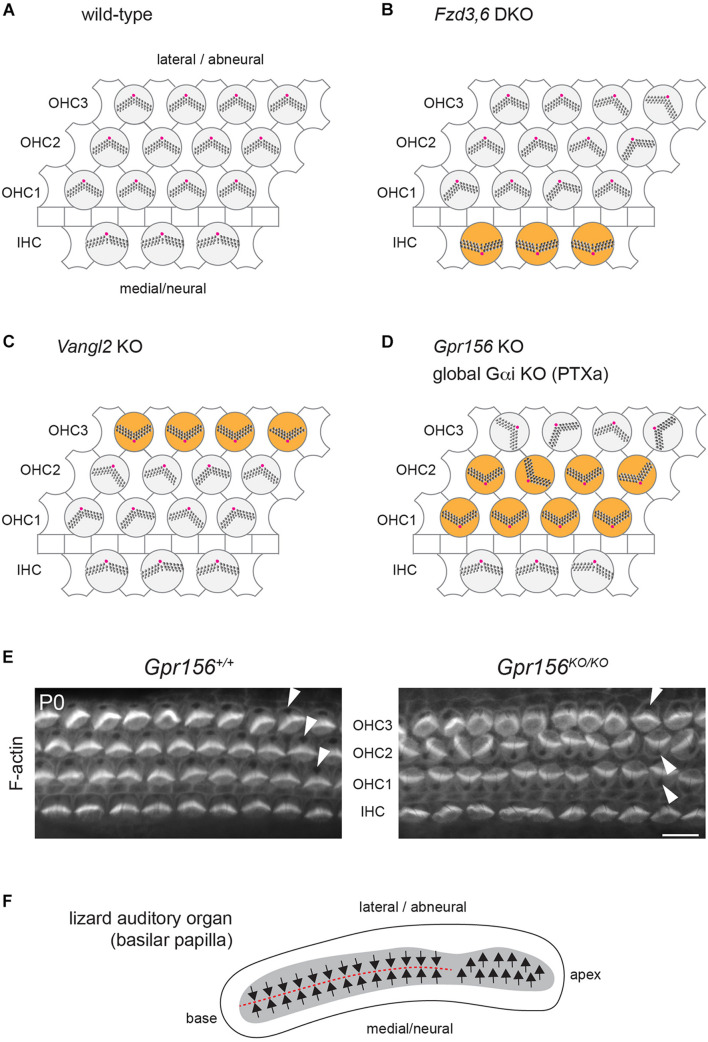
Cellular organization and hair cell orientation in the auditory epithelium of the mouse and oriental garden lizard. **(A–D)** Diagrams of the auditory epithelium in mouse neonates in a wild-type **(A)** or in mutant **(B–D)** cochleae. The off-center basal body located at the vertex of a V-shaped (outer HC, OHC) or semi-circular (inner HC, IHC) hair bundle is indicated in magenta. Note how different HC subtypes are inverted in orientation (orange) in different mutants: IHCs in *Fzd3,6* double mutants (DKO) (Wang et al., 2006), OHC3 in *Vangl2* mutants (KO) (Yin et al., 2012; Copley et al., 2013) and OHC1-2 in *Gpr156* mutants as well as upon Pertussis toxin (PTXa) expression (Kindt et al., 2021). **(E)** Conjugated phalloidin labeling of the mouse auditory epithelium at birth (P0) to reveal F-actin. F-actin is concentrated in the asymmetrical hair bundle, revealing HC orientation along with the absence of signal at the off-center basal body (arrowheads). Note how OHC1-2 are inverted in their orientation in *Gpr156* mutants. Scale bar is 10 μm. **(F)** Diagram of the basilar papilla in the oriental garden lizard after (Bagger-Sjoback and Wersall, 1973). Arrows indicate HC orientation, and the red dashed line indicates the line of polarity reversal. Note how the base of the papilla shows mirror-image HC organization reminiscent of normal macular organs and zebrafish neuromasts (Figures 2A–C), and similar to IHC and OHC1-2 orientation in *Gpr156* mouse mutants **(D,E)**.

The auditory epithelium in mammals and birds does not include mirror-image HC organization, and both IHCs and OHCs (“tall” and “short” HCs in the bird basilar papilla) are similarly oriented toward the lateral, or abneural, side of the organ (Figures 1C, 3A). Intriguingly, however, several mouse mutants have presented with a relatively precise inversion of a subset of HCs: IHCs in constitutive *Fzd3,6* double inactivation (Wang et al., 2006) and the third row of OHCs (OHC3) in *Vangl2* inactivation (Yin et al., 2012; Copley et al., 2013; Figures 3B,C).

These results are somewhat surprising because FZD3,6 and VANGL2 are transmembrane members of the core PCP family, and core PCP mutants in other systems tend to show randomization, and not inversion, of cell orientation. Randomization is expected, as core PCP proteins are generally dependent on one another for their asymmetric enrichment. A collapse of the communication system at cell-cell junctions is not expected to preserve the axis of polarity and only invert cell orientation along that axis, as reported in *Fzd3,6* and *Vangl2* mutants (the medio-lateral, or radial axis in the cochlea).

An inversion of the complementary HC types, OHC1 and OHC2, was reported in cochlear explants incubated with Pertussis toxin (Ezan et al., 2013) and in mouse models where the Pertussis toxin catalytic subunit (PTXa) is expressed in HCs *in vivo* (Tarchini et al., 2013; Tarchini et al., 2016; Figure 3D). Although these results suggest that G protein signaling inhibited by PTXa could be involved in HC orientation, they are equally difficult to interpret in isolation. First, using a bacterial toxin to downregulate functionally redundant Gαi proteins in developing HCs might produce non-physiological outcomes. Second, Gαi has other roles that are well established during HC differentiation. Gαi associates with the scaffolding regulator GPSM2 to form an atypical Gαi(GDP)-GPSM2 polarity complex that notably influences the orientation of the mitotic spindle in dividing progenitors (di Pietro et al., 2016). In post-mitotic HCs, Gαi and GPSM2 regulate cytoskeleton planar asymmetry at the bare zone, as mentioned previously (Figure 1B), and later promote stereocilia elongation with the MYO15A complex (Tarchini et al., 2016; Mauriac et al., 2017; Beer-Hammer et al., 2018; Tadenev et al., 2019).

Inactivation of the Gαi-GPSM2 complex at the bare zone is unlikely to explain the inversion of OHC1-2s observed with Pertussis toxin. First, unlike PTXa models, *Gpsm2* mutants do not show inverted OHC1-2s (Ezan et al., 2013; Tarchini et al., 2013; Bhonker et al., 2016). Second, polarized enrichment of Gαi at the apical membrane is unaffected and remains in register with the apical HC cytoskeleton in *Emx2* mutants both in macular (Jiang et al., 2017) and in zebrafish neuromast HCs (Jacobo et al., 2019). Reasoning that Gαi might play multiple roles in developing HCs, we embarked on a quest to identify alternative Gαi regulators that would strictly influence HC orientation, and not cytoskeleton morphogenesis. As Gαi is a member of the heterotrimeric Gαiβγ complex best characterized to relay GPCR signaling, we focused in particular on GPCRs highly expressed during HC differentiation. The orphan class C (glutamate) GPCR GPR156 proved to be an important missing link for auditory, vestibular and neuromast HC orientation.

In the mouse auditory epithelium, constitutive *Gpr156* mutants fully recapitulate OHC1-2 inversion observed with PTXa (Figures 3D,E), validating this defect as physiologically relevant (Kindt et al., 2021). In macular organs, *Gpr156* mutants phenocopy *Emx2* mutants, preventing EMX2-driven HC reversal in the lateral utricle and posterior saccule, and thus abolishing the LPR and mirror-image organization (Figures 2A,B). Finally, GPR156 function is highly conserved, as zebrafish *gpr156* mutants have largely unidirectional neuromasts where most HCs adopt the *emx2*-negative orientation (Kindt et al., 2021).

At the mechanistic level, unlike *Emx2*, *Gpr156* transcripts are not limited to the lateral utricle and the posterior saccule, but uniformly detected in all HCs in all inner ear sensory organs. Although *Gpr156* is thus not specifically transcribed by EMX2, EMX2 is necessary and sufficient to enrich and polarize the GPR156 protein at the apical HC junction. GPR156 is consistently detected at the junction opposite from the basal body in HCs from all organs and regions expressing *Emx2*: the auditory epithelium, the lateral utricle and the posterior saccule (Figure 2A). Additionally, the GPR156 protein is not enriched apically or polarized in HCs that do not express *Emx2*: HCs in the cristae, the medial utricle and the anterior saccule.

Genetic epistasis experiments also showed that EMX2 and GPR156 act upstream of Gαi. PTXa expression in macular organs leads to a partial or complete loss of regional HC reversal depending on the PTXa mouse strain used (Jiang et al., 2017; Kindt et al., 2021), a phenocopy of *Emx2* and *Gpr156* mutants (Figures 2A,B). PTXa can prevent forced HC reversal upon *Emx2* gain-of-function in the medial utricle, showing that Gαi functions downstream of EMX2 (Jiang et al., 2017). Furthermore, PTXa prevents EMX2-driven HC reversal without disturbing the polarized distribution of GPR156 (Kindt et al., 2021). This indicates that properly polarized GPR156 at the HC junction cannot trigger orientation reversal without Gαi function. In summary, an EMX2 > GPR156 > Gαi signaling cascade is required in HCs to trigger their reversal. GPR156 likely signals through heterotrimeric Gαiβγ proteins, as do metabotropic GABA_B_ receptors that are the closest GPR156 homologs (Kaupmann et al., 1997; Kuner et al., 1999; Robbins et al., 2001).

The distribution of core PCP proteins in the mouse maculae is not affected upon *Emx2* loss- or gain-of-function (Jiang et al., 2017). Similarly, asymmetric Vangl2 enrichment is not affected in neuromast HCs in either *emx2* or *notch* zebrafish mutants (Jacobo et al., 2019). In reciprocal experiments, *emx2* expression remains limited to half the HCs in each neuromast in *vangl2* zebrafish mutants, as in controls (Ji et al., 2018). These results suggest that the EMX2-GPR156-Gαi reversal pathway acts in parallel to core PCP. This conclusion is also supported by normal FZD6 and VANGL2 enrichment in the auditory epithelium of *Gpr156* mutants when the HC-support cell mosaic is intact (Kindt et al., 2021). In a stark departure, however, GPR156 enrichment at the medial HC junction in auditory HCs is missing or altered in *Vangl2 Looptail* mutants (Kindt et al., 2021). This suggests that GPR156 might link the core PCP pathway that regulates cell orientation at cell-cell junctions and the *Emx2* pathway that triggers a reversal of the basal body shift in HCs. In that light, GPR156 might represent one of the inferred effectors that interpret invariant core PCP patterning to make a binary decision on orientation in macular HCs (Wang et al., 2006; Deans et al., 2007). *Emx2* expression polarizes GPR156-Gαi signaling, which in turn seems to prevent the basal body from sitting nearby and prompts it to shift away instead (Figure 2A). As GPR156 overlaps in part with FZD6 at the medial HC junction in the auditory epithelium (Kindt et al., 2021), adding GPR156 in the junctional complex may turn FZD3,6 into a repulsive rather than attractive cue for the basal body. Interestingly, FZD6 is proposed to be enriched laterally in utricular HCs (Deans et al., 2007), which means that the basal body shifts toward FZD6 in *Emx2*-negative HCs (medial utricle), but away from FZD6 in *Emx2*-positive HCs that co-enrich GPR156 with FZD6 (lateral utricle, auditory epithelium; Figure 2A).

Overall, an evolutionary hypothesis is emerging where *Emx2* expression in select sensory regions of the inner ear and the neuromast triggers a reversal of HC orientation compared to a ground state of orientation defined by the core PCP framework (Jiang et al., 2017). In the vestibular system, EMX2 creates mirror-image HC organization in the maculae, distinguishing these organs from the more ancestral cristae in terms of their polarization patterns. *Emx2* expression in the auditory epithelium may be a carry-over from its local expression in the maculae. Unexpectedly, it appears that the ground state of auditory HC orientation is toward the medial/neural side of the organ, and that different effectors were recruited to globally reverse HC orientation toward the lateral side in mammals and in birds; notably, core PCP proteins for the most peripheral HC types (IHC, OHC3) and GPR156-Gαi for internal HC types (OHC1-2).

Interestingly, the lizard auditory organ (basilar papilla) does not show uniform HC orientation toward the lateral/abneural edge. Instead, the basal region of the papilla has medial HCs with a lateral orientation (like IHCs in mammals and tall HCs in birds), but lateral HCs with a medial orientation (Figure 3F; Bagger-Sjoback and Wersall, 1973; Mulroy, 1974). Normal mirror-image auditory HC organization in lizards is reminiscent of opposing IHC and OHC1-2s in *Gpr156* and PTXa mouse mutants (Figures 3D,E). The auditory organ from lepidosaurs (including lizards), archosaurs (crocodile, birds) and mammals are believed to have evolved in parallel as a pinch-off from a vestibular organ (Manley, 2000). In contrast to mammals and birds, it is possible that lizards do not have a basilar papilla fully derived from an *Emx2*-positive vestibular lineage. This may explain opposing HC orientations in lizards: specifically, lateral HCs at the papillar base might lack EMX2-GPR156-Gαi signaling and adopt a medial orientation, as mouse OHC1-2s do in the absence of *Gpr156* (Figures 3D,E).

## Some Remaining Questions and Future Directions

The link between core PCP and the EMX2-GPR156-Gαi pathway uncovered in *Vangl2 Looptail* mutants needs to be confirmed and explored further using alternative core PCP mutant models. In particular, it will be informative to test *Fzd3,6* mutants and probe for a physical interaction between FZD3,6 and GPR156 as these proteins occupy the same HC junction and are both GPCRs. Like GPR156 and FZD3,6, the adhesion GPCR CELSR1 is also enriched medially in auditory HCs, and is another core PCP candidate to interact with GPR156 (Duncan et al., 2017). Additionally, it remains unclear how, in the auditory epithelium at least, the core PCP proteins VANGL2 and FZD3,6 would both provide an early polarity framework throughout the sensory domain while also acting as effectors for orientation reversal in OHC3 (VANGL2) and IHC (FZD3,6). Perhaps different temporal roles, or different partners (late orientation reversal with GPR156?) can help explain this apparent dual activity.

It will be interesting to determine whether GPR156 activity, and thus HC reversal, depends on an agonist, possibly a secreted ligand or the extracellular domain of an integral protein in the adjacent support cell. In contrast, activation of GPR156 by EMX2 is more likely to result from polarized trafficking or polarized enrichment of the GPR156 receptor, which likely involves factors that remain to be identified. It is interesting to speculate that, unlike *Gpr156*, such factor(s) could be a direct transcriptional target of EMX2 that acts as a chaperone or binding partner to ensure that GPR156 is enriched at the apical junction and is planar polarized there. Alternatively, it is possible that EMX2 might prevent the expression of a factor that degrades GPR156 or somehow prevents GPR156 polarized enrichment. In any case, as originally predicted (Deans et al., 2007), such factor(s) would be regionally limited to one side of the LPR (*Emx2-*positive or *Emx2-*negative regions), providing an effective means to screen future candidates.

## Author Contributions

The author confirms being the sole contributor of this work and has approved it for publication.

## Conflict of Interest

The author declares that the research was conducted in the absence of any commercial or financial relationships that could be construed as a potential conflict of interest.

## Publisher’s Note

All claims expressed in this article are solely those of the authors and do not necessarily represent those of their affiliated organizations, or those of the publisher, the editors and the reviewers. Any product that may be evaluated in this article, or claim that may be made by its manufacturer, is not guaranteed or endorsed by the publisher.
